# Characterization of Y chromosome diversity in newfoundland and labrador: evidence for a structured founding population

**DOI:** 10.1038/s41431-024-01719-3

**Published:** 2024-10-29

**Authors:** Heather Zurel, Claude Bhérer, Ryan Batten, Margaret E. MacMillan, Sedat Demiriz, Sadra Mirhendi, Edmund Gilbert, Gianpiero L. Cavalleri, Richard A. Leach, Roderick E. M. Scott, Gerald Mugford, Ranjit Randhawa, Alison L. Symington, J. Claiborne Stephens, Michael S. Phillips

**Affiliations:** 1 Sequence Bioinformatics Inc., St. John’s, NL Canada; 2 Department of Human Genetics, Faculty of Medicine and Health Sciences, McGill University, Montréal, QC Canada; 3 School of Pharmacy and Biomolecular Sciences (PBS), Royal College of Surgeons in Ireland, St. Stephen’s Green, Dublin, Ireland; 4 FutureNeuro SFI Research Centre, Royal College of Surgeons in Ireland, St. Stephen’s Green, Dublin, Ireland

**Keywords:** Population genetics, Genetics research

## Abstract

The population of Newfoundland and Labrador (NL) is largely derived from settlers who migrated primarily from England and Ireland in the 1700s–1800s. Previously described as an isolated founder population, based on historical and demographic studies, data on the genetic ancestry of this population remains fragmentary. Here we describe the largest investigation of patrilineal ancestry in NL. To determine the paternal genetic structure of the population, 1,110 Y chromosomes from an NL-based cohort were analyzed using 5,761 Y-specific SNPs. We identified 160 distinct terminal haplogroups, the majority of which (71.4%) belong to the R1b haplogroup. When compared with global reference populations, the NL population haplogroup composition and frequencies primarily resemble those observed in English and Irish ancestral source populations. There is also evidence of genetic contributions from Basque, French, Portuguese, and Spanish fishermen and early settlers who frequented NL. Interestingly, the observed population structure shows geographical and religious clustering that can be associated with the settlement of the ancestral source populations from predominantly Protestant, England, and Catholic, Ireland respectively. For example, the R1b-M222 haplogroup, seen in people of Irish descent, is found clustered in the Irish-settled Southeast region of NL. The clustering and expansion of Y haplogroups in conjunction with the geographical and religious clusters illustrate that limited subsequent in-migration, geographic isolation, and societal factors have contributed to the genetic substructure of the NL population and its designation as a founder population.

## Introduction

The Canadian province of Newfoundland and Labrador (NL) is home to a population that traces its origins to the migration of European communities roughly 300 years ago. The current population is thought to be derived from approximately 25,000 immigrants in the 18th and 19th centuries who settled in remote coastal communities [1, 2]. These outports were largely isolated from each other, with little settlement in the interior of the island. Communities grew through large families but remained isolated until the 1950s with the advent of paved roads [1]. The population has continued to expand to its current size of 520,000 and, with the decline of the seafaring economy, is shifting from rural to urban centers, primarily the St. John’s metropolitan area in NL [3].

The main European ancestral source populations that settled NL were from communities around County Waterford and adjacent counties in Ireland and from the counties of Cornwall and Devon as well as fishing ports in Southern England [1, 4]. Following immigration to Newfoundland, English Protestants and Irish Catholics are thought to have remained separated by attending different schools, and rarely inter-married, further isolating these communities [5–7]. Additional European influences that are also thought to have contributed to the genetic landscape of NL are the Portuguese [8, 9], French [10], and Highland Scottish [1]. Norse settlers were present in NL for > 100 years in around 1000 A.D [11–13], although it appears that they never settled permanently. Also present in NL before, during, and after the time of European settlement were Indigenous peoples [1, 7, 14]. Since the 1900s, immigration to Newfoundland has been limited, and the genetic diversity in the province largely traces back to the original European settlers [15].

Detailed studies of Y chromosome variation have revealed male migration patterns throughout history and led to an understanding of the origins of current human populations [16–20]. These studies contributed to the development of a standardized phylogenetic tree of SNP-defined Y chromosome haplogroups maintained by the International Society of Genetic Genealogy (ISOGG) [21]. European Y chromosomes are primarily comprised of the haplogroups E, G, I, J, N, and R, with the R haplogroup comprising the majority of the Y chromosomes [22–25]. While many previous studies are limited by short tandem repeats (STRs) and/or low-resolution single nucleotide polymorphisms (SNPs) panels [22, 24–28], they provide information on the composition and frequency of major haplogroups in Europeans.

Supported by studies on the genetic structure of the population [7] and the presence of numerous rare monogenic disorders [29], the population of NL has been described as a founder population. However, information about the haplogroup composition, frequency of Y chromosome variation, and ancestral origins across NL is limited. To address these questions, the Y chromosomes of 1,110 individuals from the Newfoundland and Labrador Genome Project (NLGP) cohort [30] were analyzed in order to: (1) determine the composition and frequency of haplogroups in the paternal lineages; (2) elucidate the population structure of the Y chromosome; 3) understand how the NL population compares with the European ancestral source populations, and 4) identify evidence of founder effects based on haplogroup expansion and regional clustering.

## Materials & Methods

### Newfoundland and labrador cohort

Data from the initial 2500 participants from the NLGP study, a general population cohort from NL, was used for this analysis [30]. As part of the participants’ self-reported data, we collected information on their religion and the birthplace of their ancestors. Each participant provided a saliva sample using the DNA Genotek Oragene OG-600 collection kit (DNA Genotek, Ottawa, Canada). DNA extracted from these samples was genotyped using the Illumina Global Diversity Array (GDA; Illumina, San Diego, CA). Variant calling and quality control (QC) analysis of the genotyping data set was performed using Illumina’s Array Analysis Platform (IAAP) Command Line Interface (CLI) and GTCtoVCF pipeline (github.com/Illumina/GTCtoVCF) (Illumina, San Diego, CA). Out of the 2.1 M variants on the Illumina GDA SNP array, 5,761 SNPs on the male-specific portion of the Y chromosome were selected for analysis. QC analysis of the Y chromosome samples determined that 1110 participants (designated NLGP_1110_ cohort) had fewer than 200 missing Y chromosome calls (call rate > 96.5%).

### Phylogenetic reconstruction

The phylogenetic tree was constructed using two different methods: (1) the yHaplo software package [31], and, (2) a manual method using maximum parsimony (Supplemental Materials). Although there was concordance between the methods, the manual maximum parsimony approach gave greater resolution as it enabled the incorporation of SNPs with missing data, singleton SNPs (i.e. variation only observed in a single participant), SNPs without ISOGG designations, and the resolution of phylogenetically inconsistent SNPs (Figure S1, Table S1). Of the 5761 SNPs, 2114 were phylogenetically informative (Supplemental Material). From this set of SNPs, 160 distinct Y chromosomes were identified with unique “terminal” haplogroups. Analysis of the haplogroup frequencies and relationships to each other were used as the basis for subsequent observations and conclusions. Haplogroup Defining SNPs [21], associated with specific ISOGG long-form haplogroups, are reported whenever possible to facilitate comparison with the literature.

### Identification of descendants of NL founders

A combination of self-reported ethnicity, principal component analysis (PCA) of autosomes, and self-reported birthplaces of known paternal ancestors was used to identify individuals whose ancestors descended from early European settlers. Within the NLGP_1,110_ cohort, only 4 participants reported having Indigenous ancestry while 24 participants reported having a mixture of European and Indigenous ancestries (2.6%). Given the limited number of participants with various levels of Indigenous ancestry and the lack of an appropriate reference panel for Indigenous peoples in Eastern North America, we could not rigorously investigate the contributions of Y-DNA from Indigenous Peoples in this study. Similarly, any participants who were recent immigrants or who reported that their paternal ancestors (up to great-grandfathers) were not from NL were excluded from this analysis (see below). To assess continental ancestry, genotyping data from the autosomes of the NLGP participants was merged with autosome data from the 1000 Genomes project (1KGP3) before running PCA using PLINK 2.0 [32]. Continental ancestry was assigned using the first 5 principal components (PCs) (Figure S2).

We took two approaches to compare the NL population with potential European ancestral source populations. First, Y chromosome data from the Irish DNA Atlas [33] and the People of the British Isles (PoBI) [34] were analyzed. There were 812 SNPs that overlapped between the NLGP_1,110_ cohort and the PoBI and Irish DNA Atlas data sets; 516 were monomorphic. The remaining 296 SNPs were used to infer major haplogroup frequencies for all 856 Y chromosomes in these data sets. Second, the gnomAD allele frequency database [35] was queried. In principle, a rare variant observed in a population is more likely to be population-specific than one that is more frequent. Under this premise, all 2,114 phylogenetically informative Y-DNA SNPs identified in the NLGP_1,110_ cohort were inspected for their presence in 7 gnomAD European populations (Basque, Finnish in Finland (FIN), French, British in England and Scotland (GBR), Iberian population in Spain (IBS), Italian, and Toscani in Italia (TSI)). Of these SNPs, only 60 were observed in just one or two of these populations (Table S5). Analysis of these 60 variants was extended to all additional gnomAD populations to assess whether they were informative about potential population ancestry.

### Characterization of the NL Y chromosome population structure

**Fig. 1:**
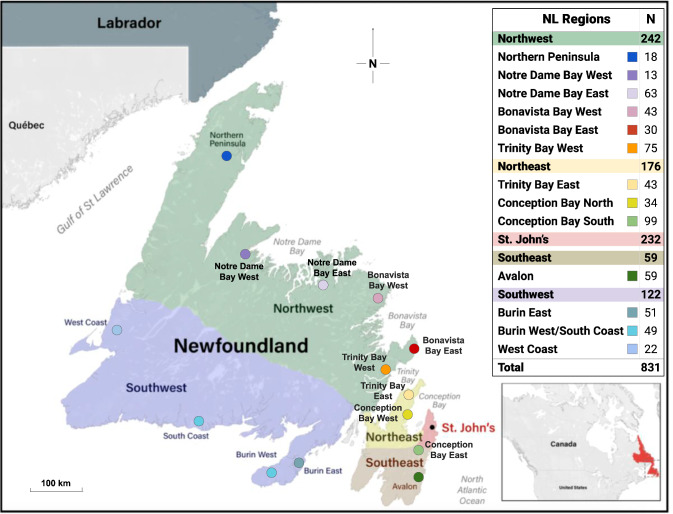
The division of Newfoundland and Labrador into geographical regions and subregions that were used for the analysis of regional Y haplogroup frequencies and composition. First-degree relatives, recent immigrants, and those missing information on the geographic location of paternal ancestors were not included.

Kinship coefficients were estimated using autosomal data and the KING relationship inference software [36] implemented in Plink2 [32]. First-degree relatives (0.177 < kinship < 0.354) were removed from population analyses. The geographical distribution of the haplogroups was mapped using the birthplace of their most distant paternal ancestor. Regions were assigned based on historical records of settlements and societal and geographic constraints. To evaluate the regional similarities and differences across NL, the province was divided into 5 large regions along the North/South axis and East/West at the point of the Avalon Peninsula isthmus. The St. John’s metropolitan area was designated as a distinct region (Fig. 1). These regions were further subdivided into 15 subregions based on major geographical features. The Labrador region, with only 4 participants, was not included in clustering analyses to avoid bias from low numbers. The remaining data set consisted of 831 individuals and 133 terminal haplogroups (designated NL_831_ cohort). Haplogroup frequencies were calculated based on geography and religion. The religious affiliation of participants was grouped into 4 categories: Catholic, Protestant, No Religion, and Other which includes all other religious/spiritual designations. Notre Dame Bay West, the Northern Peninsula, and the West Coast subregions (Fig. 1) had fewer than 25 participants which limited the interpretation of these data.

### Statistical methods

#### Haplotype diversity and FST

Haplotype diversity (H), which represents the probability that two randomly sampled Y chromosomes from a population are different, was calculated for each subregion, as well as the NL_831_ cohort as a whole (n = 831, 133 terminal haplogroups), using the following equation:

$$H=\frac{n}{n-1}\left(1-{\sum}_{i=1}^{k}{p}_{i}^{2}\right)$$

where *n* is the sample size, *k* is the number of distinct terminal haplogroups and *p*_*i*_ is the frequency of each haplogroup [37]. All distinct haplogroups were used to estimate H for the 5 major regions and 14 remaining subregions. The haplotype diversity term, H, was used to estimate F_ST_ of each subregion compared to the NL sample in R (v4.1.0) [38]. Furthermore, we computed linearized F_ST_ between each pair of regions as described by Slatkin [39] for haploid genotypes using R (v4.1.0) [38]. We used these pairwise linearized F_ST_ values to perform multidimensional scaling (MDS) analysis using the cmdscale function in R (v4.1.0) [38].

#### Statistical comparisons

To assess the stratification of paternal lineages among the 14 subregions, a PCA was performed based on the variance-covariance matrix of haplogroup frequency distribution using the PCAtools R package [40]. To determine the percentage of the variance between populations and groupings, an Analysis of MOlecular VAriance (AMOVA) was employed [41]. The percentage of variation and associated p-values were reported between populations, within populations between subregions, and within subregions. AMOVA analyses were conducted using R version 4.1.0 and packages: *ade4* package [42] obtaining simulated p-values (based on 1000 Monte Carlo simulations). For pairwise comparisons of haplogroup composition between regions and subregions, Fisher’s exact test with a simulated p-value (using 1000 Monte Carlo simulations) [43] was used with a Benjamini-Hochberg correction [44]. R version 4.0.3 was used with the *stats* package [38] to calculate p-values, and results were visualized using the *ggplot2* package [45].

## Results

### Y chromosome structure of the NL population

**Fig. 2:**
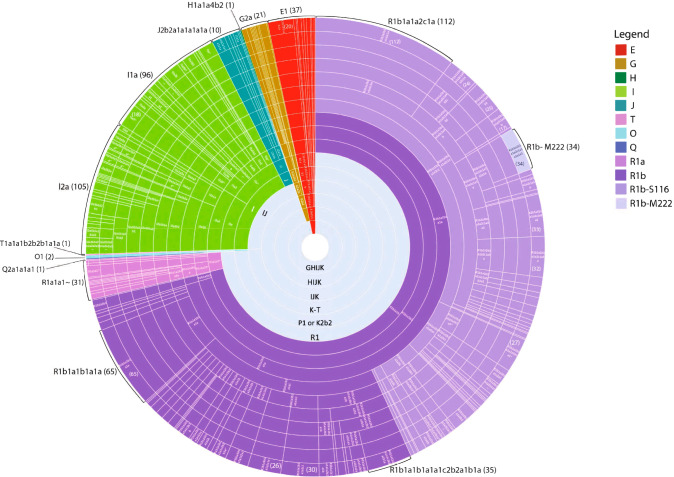
Radial diagram illustrating the proportion of individuals for each haplogroup that are present in the NLGP_1,110_ cohort. The gray inner circles represent superhaplogroups (e.g. K-T, GHIJK) in the phylogenetic tree. Each of the major haplogroups is indicated by a unique color. Each distinct terminal haplogroup is separated by a straight white line. Long-form ISOGG nomenclature is provided where possible and can be seen in greater resolution in Fig. S3. Each segment of each ring of the radial diagram is proportional to the number of Y chromosomes included in that haplogroup, and segments on the outer ring are proportional to the number of participants belonging to each terminal haplogroup. The number of participants for select terminal haplogroups are indicated by the brackets on the outer ring.

To construct the NL phylogenetic tree, we used 2114 phylogenetically informative SNPs in conjunction with the long-form haplogroup ISOGG nomenclature to assign 1110 NL participants to 160 specific haplogroups (Figs. 2 and S3, Tables 1, S2 and S3). Seventeen major internal branch points and 7 terminal haplogroups were supported by 20 or more phylogenetically informative SNPs providing confidence in the assembly of the NL Y-DNA tree (Fig. S4).

**Table 1:** List of Major Haplogroups present in the NLGP_1,110_ cohort.

Major ISOGG Haplogroup	Defining SNP	Terminal Haplogroup count	Chromosome count
E1a2a1	CTS10935	1	1
E1b1b1a1a1	V12	1	2
E1b1b1a1b1	L168	3	23
E1b1b1a1b2	L677	2	5
E1b1b1b	Z827	3	6
G2a1a1a1a1	Z6638	1	1
G2a2a1a2a1a	L166	1	6
G2a2b1	M406	1	1
G2a2b2a1	L140	6	11
G2a2b2b1a1	F872	1	2
H1a1a4b2	M2972	1	1
I1a~	CTS9857	1	12
I1a1	CTS6364	4	15
I1a2a1a1	S337	4	22
I1a2b	S296.1	2	18
I1a3~	Z63	4	29
I2a1a1	CTS595	3	18
I2a1a2	M423	2	9
I2a1b1	M223	15	64
I2a1b2a	L38	2	10
I2a2	L596	1	4
J1a	CTS5368	1	1
J2a1a	F4326	4	9
J2b2a	M241	2	13
O1a1a1	F446	1	1
O1b1a1a1a1a1	M111	1	1
Q2a1a1a1	FGC1897	1	1
R1a1a1	M417	13	31
R1b1a1b1	L23	76	791
R1b1a1b2a	GG480	1	1
T1a1a1b2b2b1a1a	CTS6507	1	1
**Total**		**160**	**1110**

The majority of the Y chromosomes in the NLGP_1,110_ cohort occur in the R haplogroup (74.2%, Table 1), predominantly within the R1b haplogroup (71.4% Fig. 2, purple). The R1b-S116 haplogroup (light purple), comprises 46 distinct haplogroups in the NLGP_1,110_ cohort (43.2%), including its subclade R1b-M222, which occurs in 3.1% of the NL Y chromosomes (Table 2). Also present are subclades of major haplogroups I2a, I1a, E1b, R1a, G2a, J2b, J2a in decreasing order of occurrence. The following 7 haplogroups, E1a, H1a, J1a, T1a, O1a, O1b, and Q2a, were detected in single participants, mostly in people who self-reported being born outside of NL.

**Table 2:** Terminal haplogroups and their relative frequencies in the NLGP_1,110_ cohort with a chromosome count of 30 or more.

ISOGG Haplogroups	Defining SNP	Chromosome Count	Frequency in NL (N = 1110)
R1b1a1b1a1a	L151	65	5.9
R1b1a1b1a1a1c1	S264	30	2.7
R1b1a1b1a1a1c2b2a1b1a	Z8	35	3.2
R1b1a1b1a1a2c1a	DF13	112	10.1
R1b1a1b1a1a2c1a1a1a1a1	M222	34	3.1
R1b1a1b1a1a2c1a3a2	CTS4466	33	3
R1b1a1b1a1a2c1a4a	Z255	32	2.9

A review of the NLGP_1,110_ cohort identified 31 terminal haplogroups present in 10 or more individuals (Table S6), and more specifically, 7 haplogroups that were present in 30 or more individuals (Table 2). The largest haplogroup was R1b-DF13 which occurred in 112 individuals.

#### Y chromosome structure of the NL population

To understand the population structure of NL, we analyzed the haplogroup frequencies by geographical region of the 831 descendants of European founders (NL_831_) (Table 3). Regional differences in R1b haplogroups are observed across NL. The R1b-S116 haplogroup represents greater than 42% of the Y chromosomes except in the Northwest region (28.9%). The frequency of the R1b-M222 haplogroup, in comparison, is highest in the Southeast region.

**Table 3:** Major haplogroup frequencies detected in the NL_831_ cohort by geographical region.

NL Region	Major Haplogroup frequencies											Number of individuals
	E1b-M215	G2a-L31	I1a-M253	I2-M438	J2a-M410	J2b-M12	R1a-M198	R1b-M269	R1b-S116	R1b-M222	T1a-M70	
Southeast	5.1	1.7	10.2	3.4	0.0	0.0	0.0	23.7	47.5	8.5	0.0	59
Northeast	1.7	1.7	6.8	8.0	0.6	1.1	2.8	30.1	43.8	3.4	0.0	176
St. John’s	2.6	1.3	6.9	7.3	0.0	1.3	2.6	28.9	47.0	2.2	0.0	232
Northwest	2.1	1.2	10.3	15.3	1.2	0.4	2.9	34.7	28.9	2.5	0.4	242
Southwest	4.1	2.5	8.2	9.8	0.8	1.6	2.5	27.9	42.6	0.0	0.0	122
**NL Cohort**	**2.6**	**1.6**	**8.3**	**9.9**	**0.6**	**1.0**	**2.5**	**30.3**	**40.4**	**2.6**	**0.1**	**831**

Each NL individual was assigned to a geographical region based on the self-reported birthplace of their most distant paternal ancestor.

Haplotype diversity based on the frequencies of 133 terminal haplogroups was used to calculate pairwise F_ST_ between the 5 major regions. The MDS plot (Figure S5) demonstrates that the major difference among populations (99.4% of the total variance) corresponds to an East-West axis of variation. Results from the AMOVA showed that most of the variation can be explained by the haplogroup distribution within subregions (99.3%; p = 0.001; described as “Within populations” in Table S7). A comparison of the Avalon subregion in the East with the Northwest subregions shows significant differences in haplogroup composition by Fisher’s exact test (p = 0.02 to 0.001) (Table S8). The East-West geographical haplogroup distribution is further supported by PC analysis as represented in the scree plots of the top 5 components (Figure S6).

**Fig. 3:**
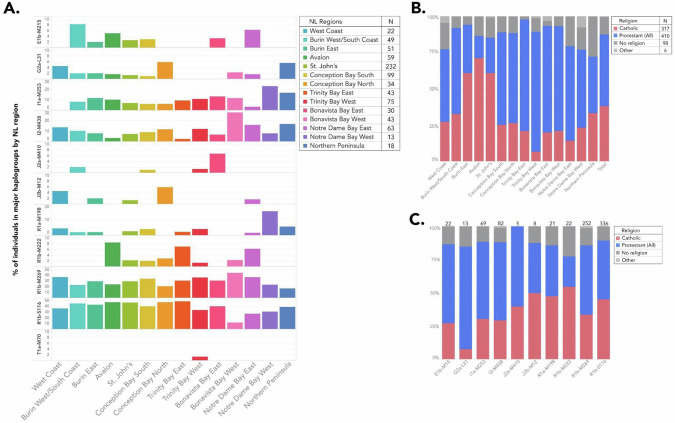
Distribution of major haplogroups by religious affiliation and geographical location. Each NL individual was assigned to a geographical region (see the position of each geographical region outlined in Fig. 1 above) based on the self-reported birthplace of their most distant paternal ancestor and religion was self-reported. **A** The frequencies and distribution of the major haplogroups represented as a percentage of the total number of individuals in that region. **B** The frequencies and distribution of self-reported religious affiliation as a percentage of the total number of individuals in a given region. **C** The frequencies of self-reported religion represented as a percentage of each major haplogroup. Only one person was reported to have a haplogroup of T1a-M70 and therefore is not represented in Fig. 3C.

The coastal communities show distinct patterns in haplogroup frequency and religious affiliation (Fig. 3A, B). The St. John’s metropolitan area has experienced immigration from many of the coastal communities. As expected, most haplogroups observed in the other regions are present in St. John’s (Figs. 3A and S7). In the Northeast region, several haplogroup frequencies differ from those observed in the overall NL_831_ cohort suggesting that these subregions might have been settled by immigrants originating from different European regions (Fig. 3A). For example, adjacent subregions on the same Peninsula in the Northeast show differential frequencies of I2-M438 ranging from 2.3% in Trinity Bay East to 11.8% in Conception Bay North (Fig. 3A). Similarly, in the adjacent Northwest region, Notre Dame Bay East, Bonavista Bay West and Bonavista Bay East subregions show significant differences in haplogroup composition when compared with subregions in both the Northeast and Southeast (p = 0.02–0.001 by Fisher’s exact test) (Fig. 3A, Table S8), further reinforcing the East-West geographical distribution of haplogroups.

Religion displays some distinctive distribution and frequency patterns across NL as previously described [1, 5–7]. Participants in the Southeast region are predominantly Catholic (>70%) while the Protestant religion predominates in the North (~ 70%) (Fig. 3B). In the NL_831_ cohort, in some regions, some haplogroups appear to be associated with a specific religious affiliation (Fig. 3C). For example, the elevated presence of I2-M438 and I1a-M253 haplogroups in the Northwest appears to be mainly associated with Protestant communities (Fig. 3C). Similarly, the R1b-M222 haplogroup, associated with Irish ancestry [28], is observed mainly in Catholic communities (Fig. 3C) and is primarily seen in the Avalon Peninsula. As Burin East is the closest subregion of the three to the Avalon subregion and closely resembles the Avalon subregion in terms of religious affiliation, it is noteworthy that R1b-M222, a haplogroup associated with catholic communities, is absent in this region (Fig. 3C).

**Fig. 4:**
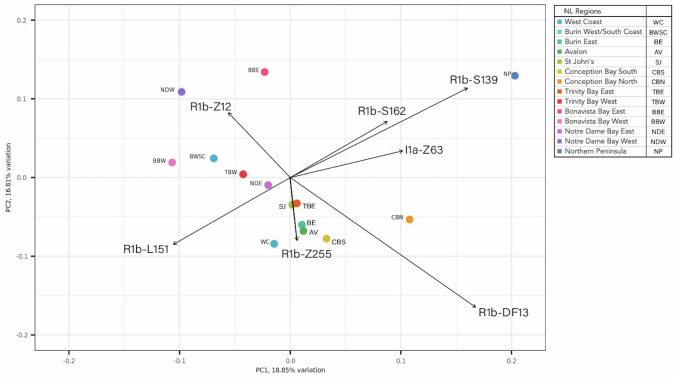
Principal Component (PC) Analysis biplot of the haplogroup frequencies in the 14 NL subregions indicating which haplogroups were the major contributors to the top 2 PC axes. Arrows represent the loadings for the 7 haplogroups whose differences in frequencies are the largest contributors to the value of the first two PCs.

A PC analysis based on 133 terminal haplogroups was used to visualize the structure of the paternal lineages in the 14 subregions of NL. The first 5 PCs explain >70% of the variation in haplogroup frequencies by subregion (Fig. S6). A biplot of the first 2 PCs (Fig. 4) identifies which haplogroups are the major contributors to the first and second dimensions of PC variation, and shows differentiation between the subregions in the Eastern and Western regions of NL. The R1b-Z255 haplogroup, which is mainly observed in Catholics (81%) in the Southeast region is the major contributor to the clustering of the populations in Eastern NL and shows a similar distribution to the R1b-M222 haplogroup. R1b-L151 and R1b-Z12 haplogroups which occur mainly in Protestant participants, located in the North Central and West Coast regions of NL, appear to be the major haplogroups that are contributing to the clustering of these populations.

### Comparison to ancestral source populations

To infer the origins of the NL paternal lineages, we compared the major Y chromosome haplogroup frequencies found in NL to those of Britain, Ireland, and other European source populations using the Irish DNA Atlas [33], PoBI [34], and the gnomAD allele frequency database [35] (Table 4). The majority of Y chromosomes (73.3%) within the PoBI and Irish DNA Atlas data sets belong to subclades of the R1b (R1b-M343) haplogroup (Table 4). In addition, analysis of the gnomAD data in combination with data from the PoBI and the Irish DNA Atlas showed evidence of specific haplogroups that could act as markers for the ancestral populations. For example, 3 R1b haplogroups, R1b-U198, R1b-L46, and R1b-Z8, seen at relatively high frequency in the NL population, are observed almost exclusively in England in the PoBI and Irish Atlas data sets and likely correspond to English paternal lines. In comparison, R1b-M222 and R1b-Z255 are seen primarily in the Catholic-dominated areas of NL and are almost exclusively seen in Irish populations. In particular, R1b-M222 comprises 23.9% of Irish Y chromosomes but only 1% of English Y chromosomes, yet ranges from 0% (Southwest) to 8.5% (Southeast) in NL (2.6% overall) (Table 4). This further supports autosomal work by Zhai et al. [7] which showed distinct clusters of Newfoundlanders with Protestant and Catholic religious affiliations [7]. The majority of other Y haplogroups seen in the British and Irish populations (subclades of E1b, I1, I2, J2a, J2b, and R1a) were also seen in the NL_831_ cohort, although at different frequencies. Given the number of applicable reference populations available, future work could be expanded to additional global reference populations to provide further insights into contributors to the NL population.

**Table 4:** Major haplogroup frequencies detected in the People of the British Isles and Irish DNA Atlas data sets sorted by geographical region.

Region	Major Haplogroup Frequencies									Number of Individuals	
	E1b-P2	G2a-L31	I1-M253	I2-M438	J2a-M410	J2b-M12	R1a-M198	R1b-M343	Other		R1b-M222
England	2.1	2.3	13.6	8.2	1.9	1.3	2.8	66.7	1.1	619	1.0
Ireland	0.9	1.7	0.9	10.3	0.0	0.9	1.7	82.9	0.9	117	23.9
Northern Ireland	0.0	10.5	0.0	15.8	0.0	0.0	0.0	73.7	0.0	19	5.3
Orkney	0.0	0.0	0.0	0.0	0.0	0.0	33.3	66.7	0.0	15	0.0
Scotland	2.4	0.0	11.9	9.5	0.0	0.0	4.8	71.4	0.0	42	7.1
Wales	0.0	2.3	0.0	4.6	0.0	0.0	2.3	88.6	2.3	44	0.0
**NL Cohort**	**2.6**	**1.6**	**8.3**	**9.9**	**0.6**	**1.0**	**2.5**	**73.3**	**0.1**	**831**	**2.6**

The frequencies of the major haplogroups were calculated and values are represented as a percentage of the total number of individuals in that region. The “Other” column represents haplogroups (F, G, N, Q and T) that were only seen once or twice in the regions (primarily the English population). The R1b-M222 haplogroup, a subclade included as part of the R1b-M343 haplogroup, is also shown separately as it represents 23.9% of the total Irish Y chromosomes.

In addition to the English and Irish ancestral populations, several other European populations are known to have fished off the coast of NL [1]. Under the premise that low-frequency variants are more likely to be population-specific, we looked for rare variants in gnomAD to identify potential source populations for Newfoundland’s founders. We identified 60 distinct variants (Table S5) that were present in only one or two of 7 gnomAD European source populations (Basque, Finnish in Finland (FIN), French, British in England and Scotland (GBR), Iberian population in Spain (IBS), Italian, and Toscani in Italia (TSI)). Further analysis of these variants was expanded to all gnomAD populations, identifying 26 distinct haplogroups. Multiple subclades of E1b, and I2a, observed 12 to 64 times in the NLGP_1,110_ cohort, were mainly found in North African and Middle Eastern populations. These haplogroups were associated, at low frequency, with the Southern European populations of France, Iberia, Basque, and Italy. It is also possible that the presence of these haplogroups in the NL population is due to the low prevalence subclades of the E1b, and I2a haplogroups in the English and Irish populations. However, the 26 specific haplogroups mentioned above were not present in PoBI or the Irish DNA Atlas cohorts. Given that the Y chromosome samples with population designations within gnomAD are limited, further study is required to validate these observations. Analysis of the gnomAD, PoBI and Irish DNA Atlas data revealed several examples of haplogroups that appear to have expanded over time in the NLGP_1,110_ cohort. For example, the R1b-L46 haplogroup which is seen in only 2 samples in the English data (PoBI), and not seen in the Irish data, appears in 14 NL participants. Likewise, R1b-Z8 is seen in 4 samples in the English data (2 samples in PoBI and 2 samples in gnomAD), but seen in 52 NLGP_1,110_ cohort participants. This observation is suggestive of both possible oversampling of specific haplogroups from England in the settlers who came to NL and possible evidence of local expansion.

## Discussion

In order to characterize the paternal lineages within NL, a high-resolution Y-DNA tree was generated using 2114 phylogenetically informative SNPs. Given the high number of Y Chromosome SNPs used in our analysis, this study represents the most detailed study of patrilineal ancestry reported to date for NL. As discussed in the methods, we did not investigate the contributions of Y-DNA from Indigenous Peoples. We recognize Indigenous peoples are under-represented in the cohort given that 2016 census data indicate 8.9% of NL self-identifies with some level of Indigenous heritage versus the 2.6% in this cohort [46]. However, to fully address the ancestral contributions of the Indigenous Peoples to the NL Y-DNA tree, a dedicated study of Indigenous Peoples, with and informed by these communities, would be warranted.

The majority of the Y-DNA haplogroups that were identified in the NLGP Y chromosomes appear to be of European origin and reside within the R1b haplogroup (71.4%). The frequency of R1b in the NL cohort is comparable to the English and Irish frequencies observed within the PoBI data, supporting both the historical records and an autosomal study by Zhai et al. [7] that immigrants from both these populations settled in NL [7]. Fine-scale population structure analysis of autosomal DNA for this dataset by Gilbert et al. [15] mirrors the results observed by the Y-DNA analysis and shows similar population clustering patterns associated with the settlement of coastal communities and the correlation with Christian denomination.

The remaining Y chromosomes in NL, primarily haplogroups I2a1 (9.9%), I1 (8.3%), E1b (2.6%), R1a (2.5%), and J (1.6%), are consistent with haplogroups that are seen in other Western European populations [23]. Many of these haplogroups have origins in specific European regions, for example, R1a and its subclades are commonly observed in Scandinavian populations [26, 47]. It is thought that much of the R1a haplogroup in England and Ireland is associated with Viking settlement [48]. As the presence of the R1a haplogroup in NL appears to reflect the frequencies seen in these data sets (Table 4), it most likely originated with the English and Irish settlers. The most prevalent of the I haplogroups in the NL_831_ cohort was I2-M438 which comprises I2a, I2b and their respective subclades. Unlike I1, the specific I2a haplogroups and its subclades that were identified in this study (I2a1ax and I2a1bx) are much less frequent in Scandinavia but are reported to comprise 10% of Irish and 6% of Basque Y haplogroups [24–26, 28]. While the presence of I2a, which is clustered in the Northwest region of the province (Fig. 3, Table 3), is consistent with English-settled communities in NL, it also could be indicative of the presence of Iberian/Basque Y-DNA that originated from Portuguese and Spanish fishermen [8, 9]. Similarly, the J haplogroup, comprising ~10% of the current Portuguese population [49] may also have originated in NL with the presence of Portuguese ancestors. These observations also strongly correlate with the autosomal chromosome clustering data [15].

Clustering patterns of haplogroups in specific communities and subregions in the NL_831_ cohort appear to be associated with clustering of self-reported religious affiliation (Fig. 3B). The observations made from this study further validate and expand on the work of Zhai et al. [7] and Gilbert et al. [15] by providing a more detailed mapping of the geographical clustering patterns of NL chromosomes. The clustering patterns of religion align with historical records of settlements in these regions, primarily Irish Catholics in the Southeast (>70% Catholic) and English Protestants in the Northwest region (70%) (Fig. 3B) [1, 4, 6, 15]. Although religious affiliation can change, our data suggest that self-reported religion in the NL population can be viewed as a surrogate marker for both religion and geographic origin of the participant’s paternal lineage in NL [4, 15]. The R1b-M222 haplogroup, a known Irish haplogroup [22, 25, 28], and R1b-Z255, speculated to be of Irish origin [50], show localized clustering to known Irish Catholic communities, specifically in the Avalon subregion (Fig. 4). Given that early migration of Irish Catholics to NL is well documented, it is likely that these settlers are the primary source of these haplogroups [1, 6]. Although the R1b-M222 haplogroup accounts for approximately 25% of Y chromosomes in Ireland [25], it is only seen at a frequency of 3% in the NL cohort. This difference is likely because the R1b-M222 haplogroup is primarily seen in Northwest Ireland [25] whereas the historical records suggest that NL was primarily settled by immigrants from Southeast Ireland [1, 25].

NL regional haplogroups exhibit differences along an East to West axis (p = 0.004; Table S7, Fig. 3, S5 & S6) and appear to be driven by the ancestral origins of the population with Irish Catholics in the South and East and English Protestants in the North and West. This observation supports the hypothesis that communities were established by settlers who originated from certain communities or specific parishes in Ireland and England and stayed isolated over time. Regions that are directly adjacent to each other, for example, Bonavista Bay East and West, only separated by ~60 km of water, show significant differences in haplogroup composition, supporting the historical records of isolation of coastal communities [4–6]. As expected, the St. John’s Metropolitan region, which has experienced recent immigration from many coastal communities, does not show the same patterns of geographical clustering. The data also indicate that there are Y chromosome contributions from additional European populations such as the Basque, Portuguese, Italian and French. All these observations support the hypothesis that paternal Y haplogroups arrived from distinct European ancestral communities to specific regions within NL.

The unique characteristics of the Y chromosome population structure in NL are indicative of a founder effect. These communities increased over the last 300 years from 25 K people to >520 K people [1–3]. Evidence of isolation and expansion can be seen in the geographical clustering patterns, and the expansion of certain haplogroups in the NL population (Table 2, Table S4). In fact, 64% of the Y chromosomes in the NLGP_1,110_ cohort show possible evidence of expansion over time as these haplogroups occur in 10 or more people (31 haplogroups in 709 people) (Table 2, Table S4). The expanded haplogroups of R1b-L151 and R1b-Z255 show evidence of regional clustering and expansion as the major haplogroups that differentiate subregions in the East (R1b-Z255) from subregions in the Northwest (R1b-L151) (Fig. 4). These observations illustrate that specific ancestral source populations from Europe settled NL, expanded over time, and contributed to the unique clustering patterns seen today. Fine-scale population structure analysis of the autosomal data support these observations and further demonstrates that the NL population is primarily comprised of a diaspora of founders from England and Ireland that settled in NL 300 years ago [15].

While this study is the most detailed of its kind, future studies of additional larger European and North American reference populations, like French Canadians and people from Acadia, may provide insight and finer detail on the migration patterns and settlement of Newfoundland and Labrador. In addition, the recent publication of the complete sequence of the Y chromosome should allow for greater resolution in future studies of Y chromosome ancestry [51]. Further comparison of historical demographic and sociological data may add additional insight into the differences seen in religious clustering in NL.

In summary, NL is an excellent example of a population exhibiting founder effects resulting from limited genetic input followed by generations of geographical and societal isolation which led to regional expansion of specific haplogroups. These data provide a better understanding of the NL genetic population structure which can inform both ancestral history and population structure.

## Supplementary information

Supplement Information

Supplemental Table S1

Supplemental Table S2

Supplemental Table S3

Supplemental Table S4

## Data Availability

The genotype and sample meta-data from the Newfoundland and Labrador Genome Project (NLGP) are not publicly available due to participant recruitment conditions and consent agreements that protect the privacy of NLGP participants. Reasonable requests for access to the genotyping data should be made to Sequence Bioinformatics. Researchers interested in accessing the NLGP data are encouraged to contact Sequence Bioinformatics (info@sequencebio.com).
