# Supplementary material for: Characterization of Y chromosome diversity in newfoundland and labrador: evidence for a structured founding population
Source: Eur J Hum Genet. 2024 Oct 29;33(1):98–107. doi: 10.1038/s41431-024-01719-3 (PMC11711158; doi:10.1038/s41431-024-01719-3)
Supplement: Supplementary file 1 — Supplement Information [file 41431_2024_1719_MOESM1_ESM.pdf]

Supplemental Information for:

Characterization of Y Chromosome Diversity in Newfoundland and Labrador: Evidence for a Structured Founding Population

Authors:
Heather Zurel[1], Claude Bhérer[2], Ryan Batten[1], Margaret E. MacMillan[1], Sedat Demiriz[1], Sadra Mirhendi[1], Edmund Gilbert[3,4], Gianpiero L. Cavalleri[3,4,] Richard A. Leach[1], Roderick E. M. Scott[1], Gerald Mugford[1], Ranjit Randhawa[1], Alison L. Symington[1], J. Claiborne Stephens[1] and Michael S. Phillips[1*]

Affiliations:
1) Sequence Bioinformatics, Inc., St. John's, Newfoundland and Labrador, Canada
2) Department of Human Genetics, Faculty of Medicine and Health Sciences, McGill University, Montréal, Québec, Canada
3) School of Pharmacy and Biomolecular Sciences (PBS), Royal College of Surgeons in Ireland, St. Stephen's Green, Dublin, Ireland
4) FutureNeuro SFI Research Centre, Royal College of Surgeons in Ireland, St. Stephen'sGreen, Dublin, Ireland

Corresponding author email:
* Michael S. Phillips: pgxdoc@gmail.com

This PDF file includes:

- Supplementary Methods: Describing the development of the NL Phylogenetic Tree
- Figures S1 to S7
- Descriptions for Tables S1 to S4 (attached as Excel files)
  - titles, column headings and descriptions of possible values
- Tables S5 to S8

Supplemental Information

Supplemental Methods

Building the phylogenetic tree

After performing the QC analysis on the genotypes of the NLGP Y chromosomes, as outlined in the methods section of the paper, we identified 5,761 SNPs that could be used to build a NL Y-DNA phylogenetic tree. For this, we employed a multi-stage analysis based on maximum parsimony (described briefly in methods and in more detail below). An overview of the process is shown in Figure S1.

We initially used the yHaplo software to assign haplogroups and construct our Y-DNA tree (https://github.com/23andMe/yhaplo) (Poznik, 2016) from our SNP genotyping dataset. While the older branches of the Y tree were confirmed, the number of phylogenetically informative SNPs available for our study suggested that building the tree manually using maximum parsimony would provide a greater level of detail than could be obtained through yHaplo.

Stage I - Identification of clades of the Y-DNA Tree using the highest quality and most complete SNP data subset.

The first stage of the analysis involved identifying all the SNPs that had 1) no missing data and 2) minor allele counts (MAC) of 4 or greater, (1,094 SNPs, Table S1). This subset of SNPs represented the most pristine part of our genotyping dataset. Using this dataset, all cohort participants were then grouped into common SNP genotype clades that contained the same SNP calls. Visual inspection of the clade groupings was performed to identify major binary splits, or “partitions”. Several major partitions were consistently identified. We chose the “M9” branch as a reference point for all clades deduced from our data. Superhaplogroup K-M9 is an ancient clade from which the major haplogroups L, T, N, O, M, P, Q, R, and S are derived (Figure S4).

Stage I analysis was based on the assumption that most of the 1094 SNPs identified above would 1) create a single binary partition or branch point for our Y chromosomes and 2) be 100% phylogenetically consistent with previous partitions. We observed that 1,072/1,094 of Stage I SNPs fell into this category, creating 100 distinct clades without any suggestion of genotyping error. The 22 remaining Stage I SNPs were identified as phylogenetically inconsistent, meaning that their genotype information conflicted with the phylogenetic inferences drawn from multiple phylogenetically consistent SNPs. Phylogenetic inconsistency can be explained by two processes, genotyping error, or genuine homoplasy (the occurrence of parallel or reverse mutations). The investigation and resolution of these inconsistencies is discussed in Stage V below, Table S3. Information on each SNP and the corresponding clade/haplogroup is provided in Table S1.

Singleton clades

Of the 100 identified clades in Stage I, there were 11 singleton clades. Singleton clades occur when a clade of "*k*" Y chromosomes is further subdivided by a SNP with MAC = *k-1*, splitting one Y chromosome off from the rest. Similarly, clades of 2 or 3 Y chromosomes were also observed in Stage I. Moreover, singleton clades were closely scrutinized and validated during Stage V when all phylogenetically inconsistent SNPs were investigated and resolved (see above and Stage V below). For the purposes of this study, we provisionally recognized these singleton clades until additional information (e.g., ISOGG labeling) could be evaluated. Once validated, the singleton clade is recognized as a singleton terminal haplogroup.

Stage II - Identification of less frequent clades of the Y-DNA Tree using SNPs with no missing data.

An additional 390/5,761 SNPs were identified as having 1) no missing data and 2) a MAC of 3 or less. Overall, these SNPs helped to further confirm clades identified in Stage I and created refinements that were all phylogenetically consistent with previously established clades. Only 3 of these SNPs were phylogenetically inconsistent, and were set aside for resolution in Stage V. Importantly, many of the 11 singleton clades from Stage I were supported by SNPs with MAC = 1, i.e. multiple singleton SNPs helped to confirm these clades. As SNPs with MAC = 1 are not traditionally used in a typical cladistic analysis, we referred to our analysis method as an "augmented" cladistic analysis. We accepted the singleton haplogroups when they met either of the following criteria:

C1) When multiple SNPs supported the singleton clade indirectly, as in the discussion below Stage I above, and/or directly as in Stages II, III, or V below;

C2) When a singleton SNP corresponded to a previously defined ISOGG haplogroup AND the ISOGG haplogroup assignment was consistent with the placement of this singleton haplogroup onto our phylogenetic tree.

Stage III - SNPs with missing data.

After completion of Stages I and II, 634/5,761 SNPs were identified as having distinct variation in addition to varying degrees of missing data (median 2 missing genotypes). These previous Stages suggested that rates of phylogenetic inconsistency due to genotyping error and/or homoplasy were relatively low (1.7%, 25/1,484 SNPs). As most of the branches in our Y chromosome tree had already been developed in Stages I and II, we were able to place 602/634 SNPs from Stage III onto the existing tree as the missing data was often irrelevant to clade assignment. For example, if the associated clade suggested by the SNP data was deep within M9+ chromosomes, and the missing data was only among M9- chromosomes, there was no difficulty evaluating whether the SNP reinforced an existing, or even defined a new, M9+ clade. In several cases entirely new branches were identified, and these were nonetheless phylogenetically consistent with existing clades.

As expected, Stage III SNPs had a higher frequency of phylogenetic inconsistency (5%, 31/634) than SNPs identified in the previous two Stages of analysis. Additionally, 109/634 (17%) of the SNPs required further review to place on the NL Y-DNA tree as missing data

confounded clade assignment. However, even with missing data, most of the missing genotypes could be inferred (imputed) by reliance on downstream SNPs that further refined the haplogroup assignment and many of these SNPs also had ISOGG labels. In addition to providing support for many of the provisional singleton haplogroups, the ISOGG nomenclature often enabled clade imputation in cases of missing data.

Stage IV - Monomorphic SNPs

For our Stage IV analysis, 3,643/5,761 SNPs were identified as monomorphic and several also had varying degrees of missing data. Monomorphic SNPs with ISOGG labels (see below) proved to be very valuable for identifying Y chromosome lineages that were absent in our NL cohort (Table S1). Occasionally, a monomorphic SNP had an ISOGG label for a haplogroup that was present in our dataset and these are flagged in Table S1.

Stage V - Incorporation of ISOGG Long-Form Nomenclature, Haplogroup Assignment, and Reconciliation of Phylogenetically Inconsistent SNPs

In Stage V, the SNPs associated with each clade were manually reviewed and long-form haplogroups were unambiguously assigned to all clades and branch points up to the most highly defined subclade possible (see detailed phylogenetic tree in Figure S3). When a SNP marker identified a new haplogroup that was not reflected in the current ISOGG nomenclature, the new haplotypic extension was annotated with a caret (^). In several cases, such haplogroup extensions were clustered in the same haplogroup. To address this, we annotated the ISOGG long-form root haplogroup with ^, ^^, ^^^, accordingly.

As shown in Table S1, 995 SNPs in Stage I, 302 in Stage II, 515 in Stage III, and 1,988 in Stage IV had an ISOGG designation. Notably, 300 of the 2,114 phylogenetically informative SNPs in Stages I - III have not yet been given an ISOGG label.

At a phylogenetic level, the ISOGG standardized nomenclature proved to be enormously useful in providing additional support for:

1. The confirmation of all identified clades including the singleton haplogroups,
2. The ordering of the various multifurcations arising from maximum parsimony analysis,
3. The confirmation of clades that are absent in the NL cohort, and
4. Resolution of the 56 phylogenetically inconsistent SNPs (see below).

In Stages I-III, 56 SNPs were identified as phylogenetically inconsistent with the NL Y-DNA tree and were reviewed to assess genotyping error and/or homoplasy (see section on Genotyping Error and Homoplasy below and Figure S1). The subsequent analysis of the 56 SNPs resulted in 53 SNPs being recovered and 3 SNPs that could not be reconciled. The 3 SNPs that could not be reconciled were removed from further analysis. Therefore, in total, 2,114 Y chromosome SNPs were found to be phylogenetically informative and were used for the construction of the phylogenetic tree for the NLGP Y chromosomes.

The agreement between the NL Y-DNA tree with the consensus ISOGG phylogenetic tree and long-form labels was outstanding. After review, only 29/3,800 (0.76%) of the ISOGG-labeled SNPs showed any type of discrepancy between our branch placement and the ISOGG labels. Of these 29 SNPs, 9 were phylogenetically informative and consistent with a single clade, 11 were phylogenetically inconsistent with clades emerging from each SNP's resolution either rejected as errors or discrepant with the ISOGG label when accepted, and 9 SNPs were singletons rejected as likely genotyping error (NU in Tables S1 and S3). An additional 22 SNPs were monomorphic in our dataset but whose ISOGG labels nonetheless corresponded to haplogroups present in NL. Upon inspection of each of these, 18 SNPs were missing data for the critical individuals. Since the remaining 4 monomorphic SNPs had discrepancies between our observed clades and their ISOGG labels, we observed a total of 33/3,800 SNPs (0.87%) in which the ISOGG labels were discrepant from our clade assignments.

Genotyping Error and Homoplasy

During the process of developing the NL phylogenetic tree (Figure S1), 56 SNPs were identified as phylogenetically inconsistent (Table S3). Phylogenetic inconsistency can be explained by two processes, genotyping error, or genuine homoplasy (the occurrence of parallel or reverse mutations). Our resolution of such SNPs assumes that there is an underlying phylogenetic signal, but that this signal has been confounded by secondary changes (homoplasy, H) or by genotyping error (E), or even by both (H+E). These sources of inconsistency can be differentiated, to a degree, by assuming that most genotyping error is random, and that random error is likely to generate a singleton call, that is, differentiating one Y chromosome from the others in an otherwise homogeneous clade. Since our genotypes are strictly binary, an erroneous singleton call amounts to either the generation of a singleton clade (see above), or, more often, to that Y chromosome being assigned to a haplogroup well outside of its consensus placement. Utilizing these principles, we were able to resolve 53/56 phylogenetically inconsistent SNPs identified in our maximum parsimony analysis into 24 that can be attributed to genotyping error (E), 16 that can be explained as homoplasy (H), and 13 that could be explained by homoplasy plus genotyping error (H+E). As mentioned above, the remaining 3 inconsistent SNPs that could not be resolved were not used.

From this process of resolution of the 53 phylogenetically inconsistent SNPs, 13 apparent genotyping errors were identified in our dataset. Another 37 singleton calls resulting from this resolution were rejected as probable genotyping errors, since there was no additional support as singleton clades (see C1 and C2 in Stage II). Similarly, one singleton clade from Stage I and 18 singleton SNPs from Stage II had no additional support and were rejected as potential genotyping errors. Adding to these 69 inferred errors the 14 heterozygous genotypes observed (also assumed to be erroneous), we estimate a genotyping error rate of 0.0035% (83/2,341,277). This illustrates the high quality of our genotyping data.

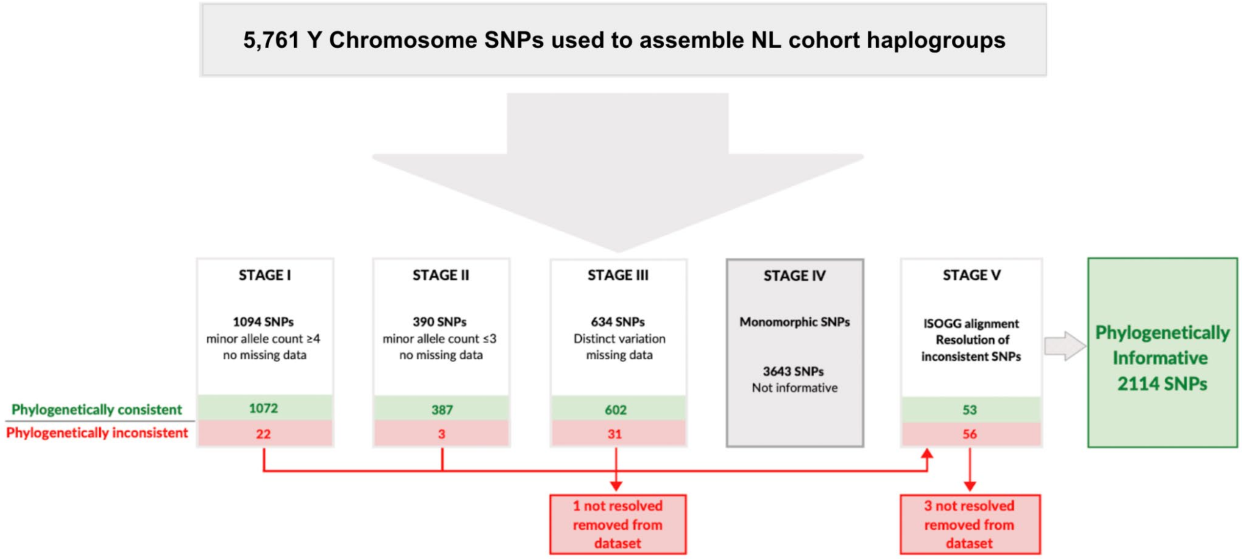

Figure S1. Analysis Method used for identifying phylogenetically informative Y-DNA SNPs in the NL cohort. This analysis uses a multistage maximum parsimony approach to identify both phylogenetically informative and inconsistent markers. In stage V, all SNPs were aligned and compared with known ISOGG haplogroups to finalize the phylogenetic tree and resolve inconsistencies.

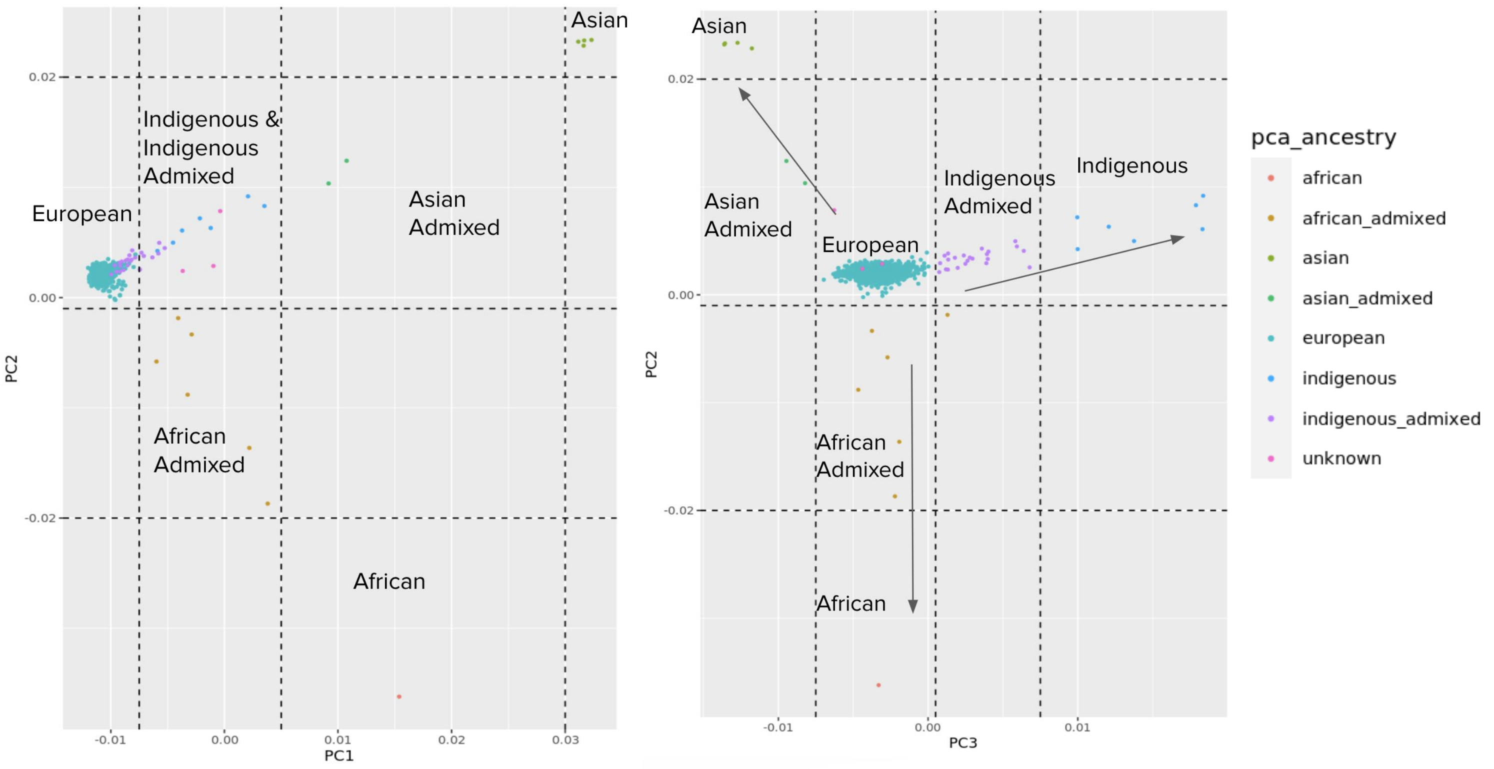

Figure S2. Principal Component (PC) Analysis of the NL Cohort (males and females). a) PC1 vs PC2, b) PC3 vs PC2. NLGP samples were merged with 1000 Genomes samples, followed by the PC analysis using Plink2. The first 3 PCs are plotted only for NLGP samples, and coloured by PC-derived continental ancestry. The PC analyses were used to help identify individuals who are descendants of the original settlers to NL. Geographic origins of outliers is based on self-reported ethnicity (i.e. PC1 and PC2 outliers have self-reported African and Asian ancestry or admixture).

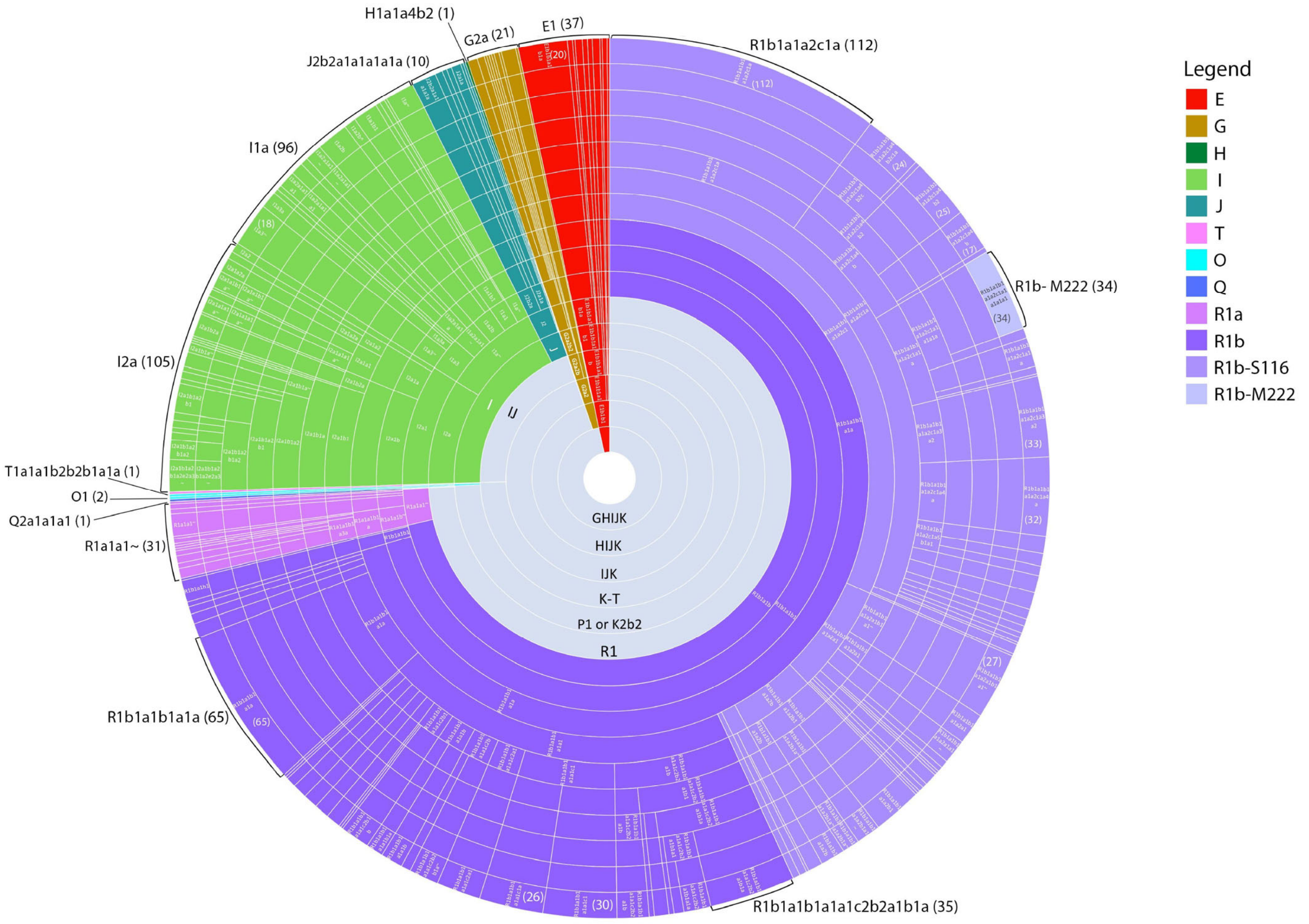

Figure S3. High Resolution Radial diagram illustrating the proportion of individuals for each haplogroup that are present in the NLGP$_{1,110}$ cohort. The gray inner circles represent mega haplogroups (e.g. K-T, GHIJK) in the phylogenetic tree. Each of the major haplogroups is indicated by a unique color. Each distinct terminal haplogroup is separated by a straight white line. Long-form ISOGG nomenclature is provided where possible. Each segment of each ring of the radial diagram is proportional to the number of Y chromosomes included in that haplogroup, and segments on the outer ring are proportional to the number of participants belonging to each terminal haplogroup. The numbers of participants for select terminal haplogroups are indicated by the brackets on the outer ring.

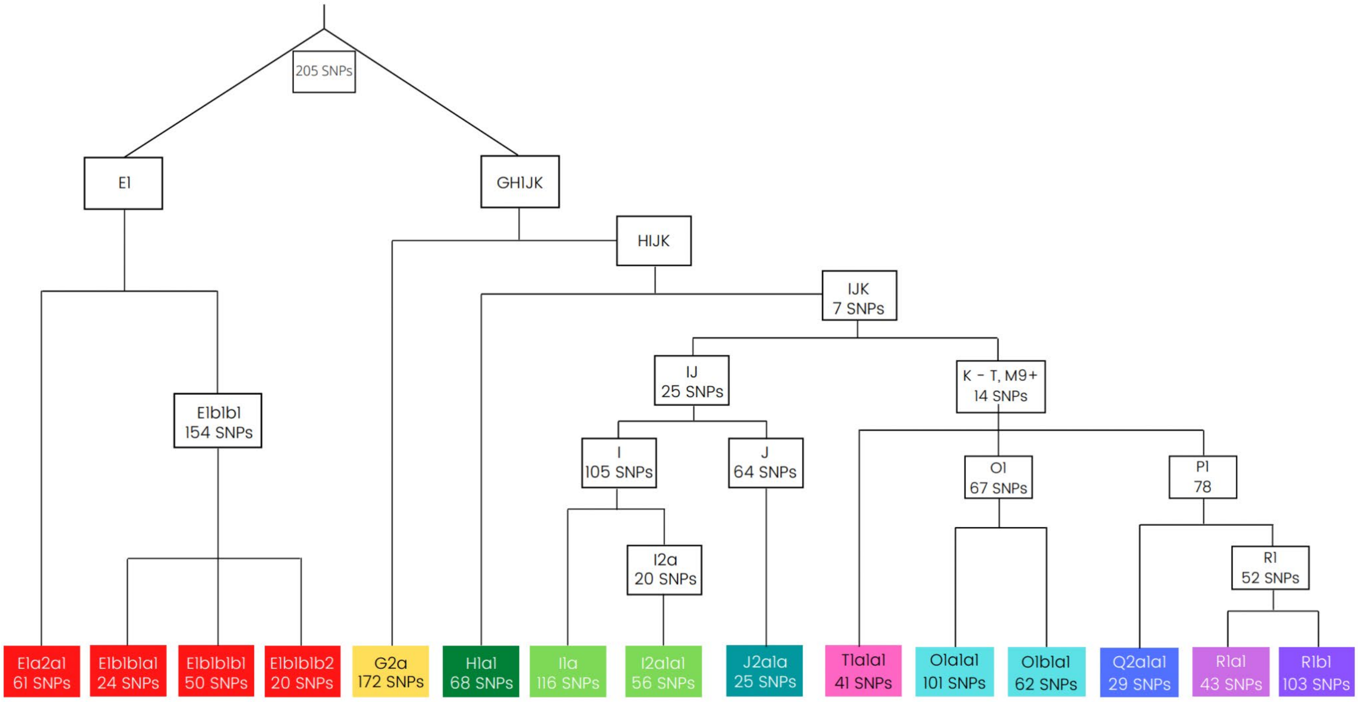

Figure S4. Schematic diagram illustrating the 17 major internal branch points and 7 terminal haplogroups that are supported by 20 or more phylogenetically informative SNPs. This figure illustrates the breadth of SNP content that was available to develop the NL cohort Y chromosome phylogenetic tree for the 1,110 participants.

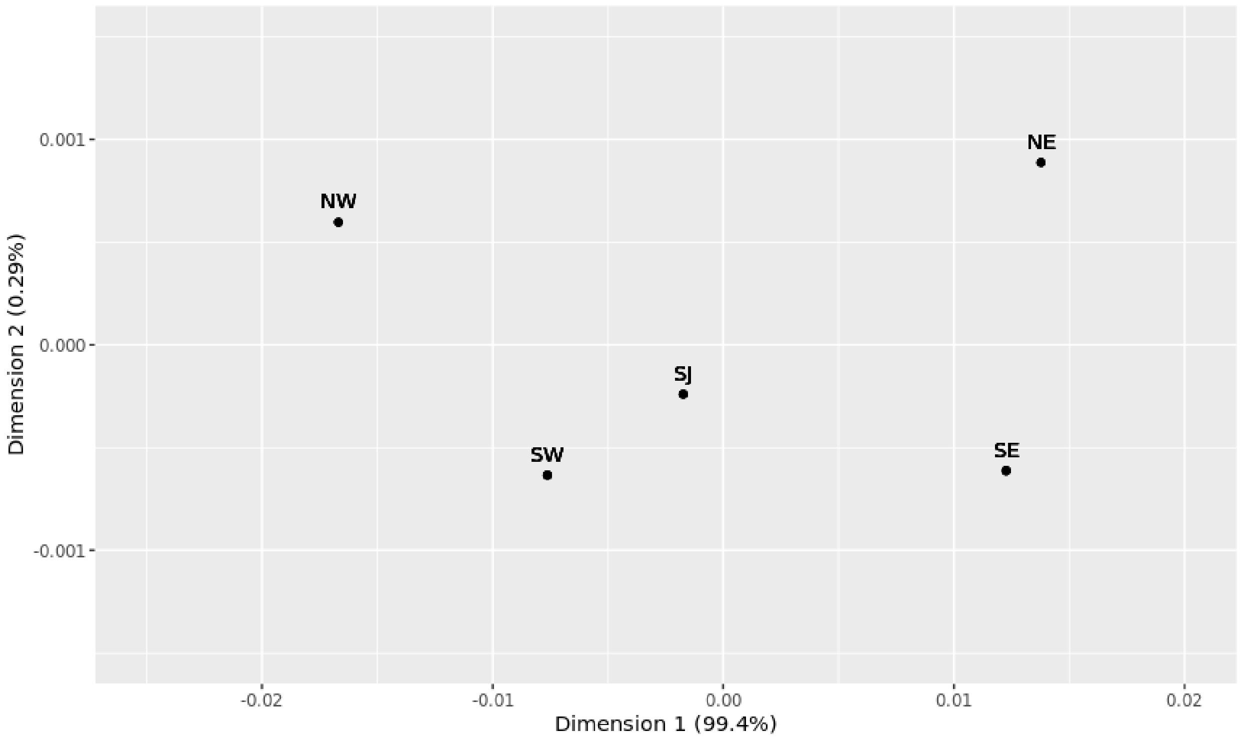

Figure S5. MDS visualization of pairwise F_{ST} values for 5 major regions in NL. The plot was generated for the 5 major regions (Southeast (SE), Southwest (SW), St John's (SJ), Northwest (NW) and Northeast (NE)) using F_{ST} values calculated with the 133 haplogroups of the 831 participants that were identified as likely descendants of NL settlers. Values of Dimension 1 were multiplied by -1 so Western regions appear on the left of the plot.

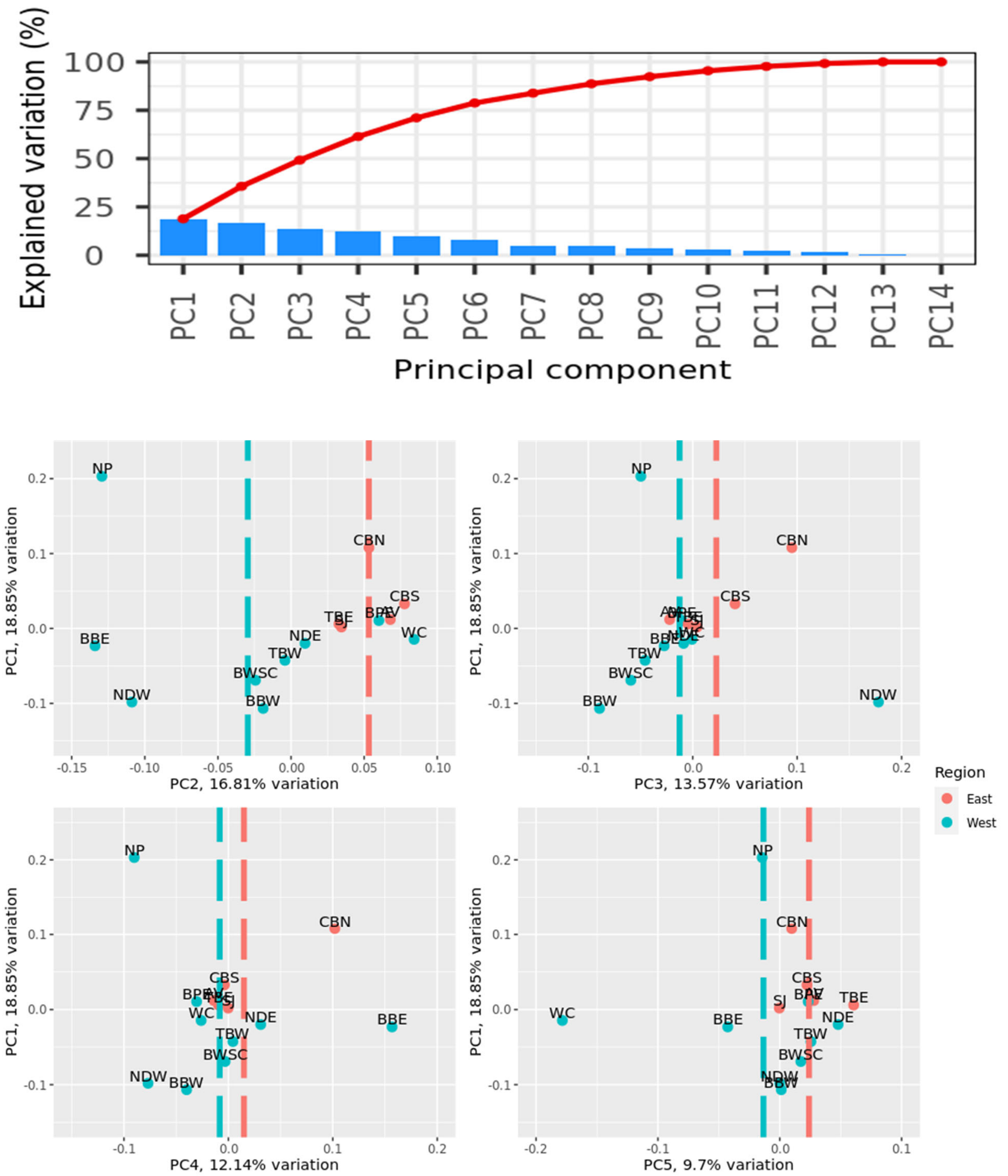

Figure S6. Principal Components (PC) Analysis of Y chromosome haplogroups in the NL cohort (n = 831). Top: Scree plot showing the proportion of variation explained by each PC. Bottom: PC1 vs PC2-PC5, colored by major region in NL. Vertical lines indicate the mean value of that PC for Eastern vs Western subregions. Note: values of PC2 in the middle-left plot are multiplied by -1 so the western subregions always appear on the left.

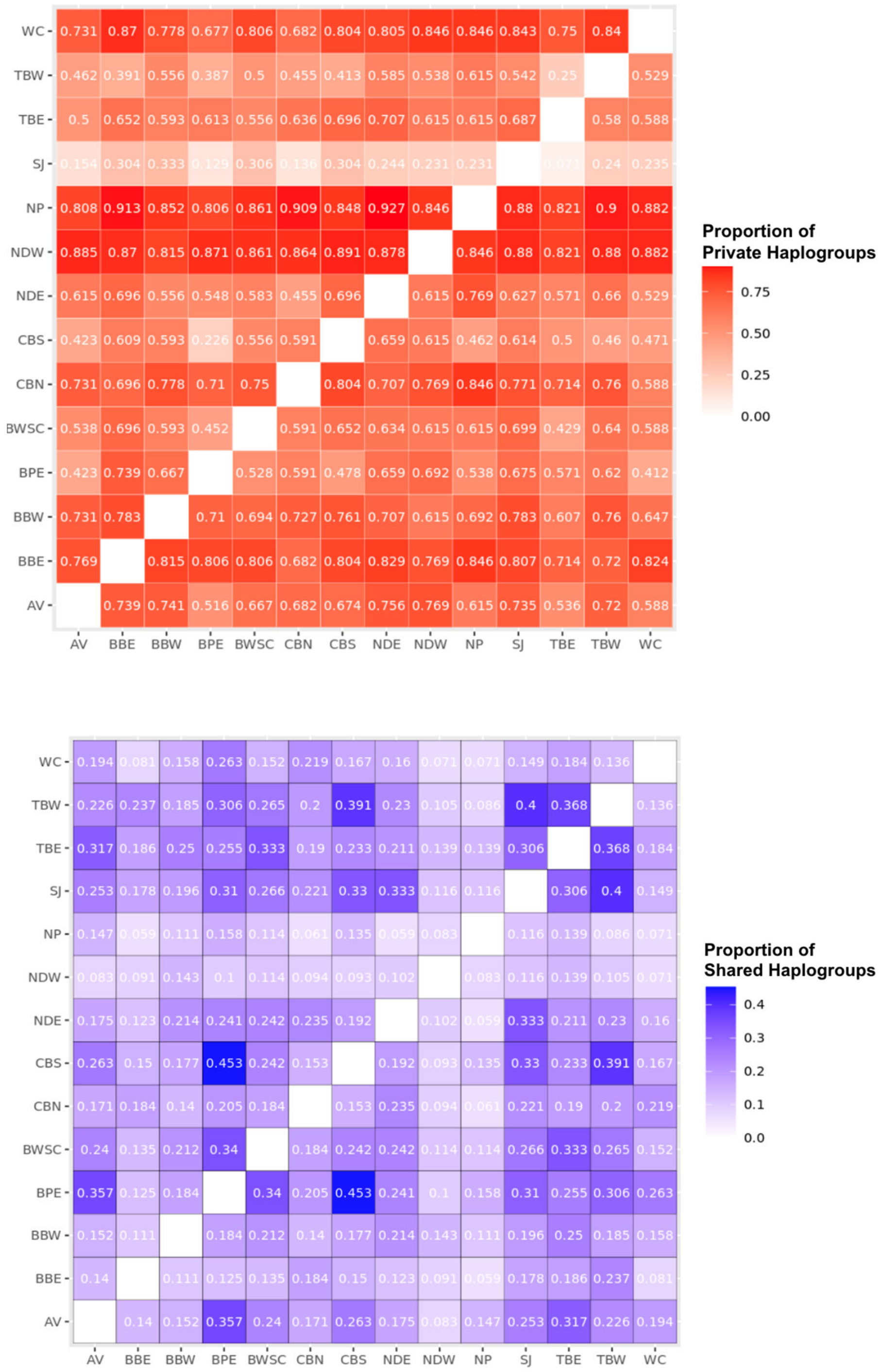

Figure S7. Heatmaps of the proportion of private and shared haplogroups in 14 subregions in NL. Private haplogroups are those that are seen in one region, but not in the other region they are being compared to. Shared haplogroups are those that are present in both subregions. As this is a pairwise comparison between two specific subregions, private haplogroups reported here could be present in the remainder of the cohort.

Supplemental Tables

Tables S1-S4 are provided in Excel format. Tables S5-S7 are below.

Table S1. Complete SNP Table. Results of analysis and phylogenetic reconstruction for all 5761 SNPs. Column headings and a description of possible values are as follows:

- List -- Numerical, ordered by Build 37 Position.
- Stage -- I through V
- M9+/M9- (0, 1,) -- these seven columns are the summary genotype data (counts) for each SNP.
- M9? -- M9- are clades splitting off prior to M9; M9+ are those after M9; M9-/M9+ are the M9 split itself; Mono are monomorphic SNPs; phylogenetically inconsistent (PI) SNPs with M9- and M9+ variability are labeled BOTH.
- Clades -- Simple numerical designation of terminal branches or clades, essentially following alphabetical order given by inferred ISOGG Subgroup name. 1 -- 66 for M9- Clades, 1 -- 94 for M9+ Clades. NU: not used, for SNPs in which a single genotyping error was deemed more likely than the clade suggested.
- Prob. -- Problematic SNPs. PI -- phylogenetically inconsistent (56 SNPs); S -- singleton SNPs without additional support, inferred to be genotyping errors; 1H = one heterozygous genotype (13 SNPs), or 2H = heterozygous genotypes (1 SNP), converted to missing data; MD = missing relevant data
- Build 37 Position -- base pair position of SNP according to GRCh37
- Name -- ISOGG Haplogroup Defining SNP name
- ISOGG Subgroup Name -- ISOGG long-form haplogroup name
- Amb. -- ISOGG label ambiguity: A = multiple distinct ISOGG labels assigned; W = wrong for our data

Table S2. Haplogroup Assignments in the NL Cohort. All clades and their corresponding ISOGG long-form haplogroup assignments identified in the NL cohort are reported, with counts in the entire cohort (n = 1,110) and for descendants of likely NL founders (n = 831).

Table S3. Phylogenetically Inconsistent SNPs. Summary data on phylogenetically inconsistent SNPs and their resolution. Column headings and a description of possible values are as follows:

- List -- Numerical, ordered by GRCh37 Position.
- Stage -- I through V
- M9+/M9- (0, 1,.) -- these seven columns are the genotype data for each SNP.
- M9? -- as in Supplementary Table 1, M9- are clades splitting off prior to M9; M9+ are those after M9. BOTH, in the first line summary for each SNP, indicates that variation on both sides of M9 was evaluated for potential clades.
- Clades -- as in Supplementary Table 1, simple numerical designation of terminal branches or clades, essentially following alphabetical order given by inferred ISOGG Subgroup name. 1 -- 66 for M9- Clades, 1 -- 94 for M9+ Clades.

- Res. -- PI SNP Resolution: PI (phylogenetically inconsistent) in each SNP's summary line, followed by one or more of: H (homoplasy), 1E or 2E (1 or 2 genotyping errors predicted), S (flag for singleton calls), MD = missing relevant data, BAD (as above in Clades column).
- Build 37 Position -- base pair position of SNP according to GRCh37 (suppressed in extensions).
- Name -- SNP's identifier (suppressed in extensions).
- ISOGG Subgroup Name -- Subgroup Name assigned to each SNP, when available
- Amb. -- ISOGG label ambiguity: A for multiple distinct ISOGG labels available, W for wrong for our data (see Supplementary Table 3).
- List -- Numerical, ordered by GRCh37 Position. Should be identical to the first column.

Table S4. Reconstructed phylogenetic tree of NLGP participants. All clades and branch points are indicated, as well as the number of participants in each clade and the ISOGG long-form haplogroup associated with each clade.

Table S5. Y-DNA SNPs identified in the $NLGP_{1,110}$ cohort that were present in just one or two of 6 known gnomAD European Settler Populations. Finns were included as a control group. The 7 gnomAD populations that were studied were Italian, British in England and Scotland
(GBR), French, Iberian population in Spain (IBS), Toscani in Italia (TSI)), Basque, and Finnish in Finland (FIN), Only 60 SNPs were observed in just one or two of these populations

	Illumina Probe Name	Y Chromosome Position	Select gnomAD Populations						
			italian	gbr	french	ibs	tsi	basque	fin
1	ilmnseq_rs551475553	2749995	0	0	0	0	1	0	0
2	200610-389	6814246	0	0	1	0	1	0	0
3	200610-300	6842263	0	0	1	0	3	0	0
4	exm1667582	6931957	0	0	0	0	1	0	0
5	ilmnseq_rs369606755	6994764	0	0	0	1	3	0	0
6	ilmnseq_rs530705466	7598950	0	0	0	0	1	0	0
7	ilmnseq_rs376788389	8015672	0	0	0	1	3	0	0
8	ilmnseq_rs558309279	8262442	0	0	1	0	2	0	0
9	ilmnseq_rs547817702	8387539	0	1	0	0	0	0	0
10	ilmnseq_rs372687543	8467290	0	0	0	1	3	0	0
11	ilmnseq_rs558848001	8483852	0	0	0	0	4	1	0
12	ilmnseq_rs566649000	8519704	0	0	0	0	1	0	0
13	ilmnseq_rs371720608_F2BT	8628308	0	0	0	0	2	0	0
14	ilmnseq_rs560134074	9778243	0	0	1	0	0	0	0
15	ilmnseq_rs549191151	14002366	0	0	0	0	1	0	0
16	ilmnseq_rs535292085	14116322	0	0	0	1	2	0	0
17	exm-rs35248080	14286528	0	0	0	3	3	0	0
18	ilmnseq_rs559633243	14645286	0	0	0	0	0	1	0
19	exm1667677	14898163	0	0	0	0	3	0	0
20	exm1667733	15029413	0	2	0	0	0	0	0
21	ilmnseq_rs571240053	15040454	0	0	0	0	0	1	0
22	exm-rs7892889	15668070	1	0	0	0	0	0	0
23	ilmnseq_rs537945911	15752242	0	0	0	0	1	0	0
24	ilmnseq_rs535347318	16327939	0	0	0	0	1	0	0
25	exm-rs17222279	16839499	0	0	1	0	0	0	0
26	ilmnseq_rs376481998	17173391	0	0	0	1	3	0	0
27	ilmnseq_rs560330907	17476068	0	0	0	2	2	0	0
28	ilmnseq_rs576940616	17476211	0	0	1	0	0	0	0
29	ilmnseq_rs543507836	17800761	0	0	1	0	0	0	0
30	ilmnseq_rs538408703	17887908	0	0	1	0	0	0	0
31	ilmnseq_rs527438348	17941203	0	0	1	0	0	0	0
32	exm-rs1469371	18167479	0	0	0	12	0	4	0
33	ilmnseq_rs577725980	18182848	0	0	1	0	0	0	0
34	ilmnseq_rs556721115	18207718	0	0	1	0	0	0	0
35	ilmnseq_rs537690249	18670504	0	0	0	11	0	3	0
36	ilmnseq_rs528767521	18776848	0	0	0	0	0	1	0
37	ilmnseq_rs552207635	18788668	0	0	1	0	0	0	0
38	ilmnseq_rs571244308	18823705	0	1	0	0	0	0	0
39	exm-rs13304625	19077409	0	1	0	0	0	0	0
40	ilmnseq_rs547345637	19087143	0	0	0	0	1	0	0
41	ilmnseq_rs529224675	19276174	0	3	0	0	0	0	1
42	ilmnseq_rs533877526	19349905	0	0	0	0	1	0	0
43	ilmnseq_rs376037006	19383622	0	0	0	1	0	0	1
44	ilmnseq_rs570146722	19513372	0	0	0	0	1	0	0
45	ilmnseq_rs547132672	19535440	0	0	1	0	0	0	0
46	ilmnseq_rs540339958	19563853	0	0	1	0	0	0	0
47	ilmnseq_rs371942647	21406133	0	0	0	1	3	0	0
48	200610-219	21717307	0	0	0	2	0	0	0
49	ilmnseq_rs529210236	21827794	0	0	0	4	0	5	0
50	exm-rs2032629	21865821	0	0	0	2	0	0	0
51	exm-rs2032648	21904023	0	0	1	0	4	0	0
52	exm-rs34283263	22178569	0	2	0	0	0	0	0
53	ilmnseq_rs575963070	22951796	0	0	1	0	0	0	0
54	ilmnseq_rs564450276	23033176	0	0	1	0	0	0	0
55	ilmnseq_rs565686288	23059496	0	0	0	2	2	0	0
56	ilmnseq_rs575331280	23327970	0	0	0	0	1	0	0
57	ilmnseq_rs371568410	23454053	0	0	0	1	3	0	0
58	ilmnseq_rs576906965	23956870	0	0	0	1	2	0	0
59	ilmnseq_rs539007522	23976740	0	0	0	3	0	0	0
60	ilmnseq_rs372722976	23977118	0	1	0	0	1	0	0

Table S6. Terminal haplogroups present in more than 10 participants in the NL cohort (n = 1,110).

Terminal haplogroups with chromosome count > 10					
M9-			M9+		
**Clade**	**ISOGG**	**Count**	**Clade**	**ISOGG**	**Count**
4	E1b1b1a1b1a	20	18	R1b1a1b1a1a	65
22	I1a~	12	28	R1b1a1b1a1a1c1	30
24	I1a1b1	10	29	R1b1a1b1a1a1c1a	26
31	I1a2b	13	30	R1b1a1b1a1a1c2a1	18
33	I1a3~	18	33	R1b1a1b1a1a1c2b1b	12
49	I2a1b1a2b1	12	37	R1b1a1b1a1a1c2b2a1b	13
56	I2a1b1a2b1a2e2a3~	13	39	R1b1a1b1a1a1c2b2a1b1a	35
66	J2b2a1a1a1a1a	10	42	R1b1a1b1a1a1c2b2a1b1a1a	11
			45	R1b1a1b1a1a1c2b2b1a~	12
			47	R1b1a1b1a1a2a1	13
			50	R1b1a1b1a1a2a1a1~	10
			58	R1b1a1b1a1a2a1b1a1~	27
			60	R1b1a1b1a1a2b	10
			61	R1b1a1b1a1a2b1	20
			63	R1b1a1b1a1a2b1a1~	10
			66	R1b1a1b1a1a2c1a	112
			70	R1b1a1b1a1a2c1a1a	12
			74	R1b1a1b1a1a2c1a1a1a1a1	34
			78	R1b1a1b1a1a2c1a3a2	33
			79	R1b1a1b1a1a2c1a4a	32
			80	R1b1a1b1a1a2c1a4b	17
			82	R1b1a1b1a1a2c1a4b2	25
			84	R1b1a1b1a1a2c1a4b2c1a	24

Table S7. AMOVA calculated with an equidistant matrix of 133 terminal haplogroup frequencies in 14 subregions in NL. Proportion of variance explained by populations, subregions, and within subregions is reported, as well as p-values based on 999 simulations (n = 831). The Eastern region contains all subregions in the Northeast, southeast, and St. John's regions and the Western regions include all subregions within the Northwest and Southwest regions. Subpopulations/subregions within these groupings are defined in Figure 1. Significant values have been bolded.

Regional Groupings	% Total variance	p-value
Regional analysis (East & West)		
Within populations	99.3	**0.001**
Among populations within groups	0.40	0.004
Among groups	0.26	0.019
Regional analysis (NE, SE, SJ, NW, SW)		
Within populations	99.43	**0.002**
Among populations within groups	0.44	0.016
Among groups	0.13	0.137
Subregional analysis (14 subregions)		
Within populations	99.45	0.005
Among populations	0.55	0.005

Table S8. Fisher's exact test with 1000 simulations (n = 831).

NL Region		Northern Peninsula	Notre Dame Bay West	Notre Dame Bay East	Bonavista Bay West	Bonavista Bay East	Trinity Bay West	Trinity Bay East	Conception Bay South	Conception Bay North	St. John's	Avalon	Burin East	Burin West South Coast
	N	18	13	63	43	30	75	43	99	34	232	59	51	49
West Coast	22	0.083	0.441	0.209	0.045	0.024	0.515	0.321	0.129	0.199	0.698	0.049	0.558	0.317
Burin West South Coast	49	0.086	0.500	0.354	0.109	0.100	0.852	0.726	0.001	0.011	0.033	0.013	0.661	
Burin East	51	0.153	0.140	0.129	0.043	0.001	0.987	0.584	0.902	0.091	0.722	0.161		
Avalon	59	0.009	0.005	0.010	0.001	0.001	0.018	0.103	0.012	0.002	0.933			
St. John's	232	0.099	0.084	0.013	0.002	0.001	0.075	0.965	0.128	0.471				
Conception Bay North	34	0.049	0.096	0.423	0.002	0.051	0.167	0.138	0.049					
Conception Bay South	99	0.008	0.041	0.002	0.001	0.001	0.055	0.037						
Trinity Bay East	43	0.144	0.350	0.705	0.070	0.009	0.996							
Trinity Bay West	75	0.147	0.363	0.312	0.458	0.123								
Bonavista Bay East	30	0.045	0.333	0.006	0.001									
Bonavista Bay West	43	0.002	0.108	0.029										
Notre Dame Bay East	63	0.028	0.271											
Notre Dame Bay West	13	0.239												
